# Personalized Radioproteomics: Identification of a Protein Biomarker Signature for Preemptive Rescue by Tocopherol Succinate in CD34^+^ Irradiated Progenitor Cells Isolated from a Healthy Control Donor

**DOI:** 10.4172/jpb.1000349

**Published:** 2015-01-28

**Authors:** Anjali Srivastava, Ximena Leighton, Ofer Eidelman, Joshua Starr, Catherine Jozwik, Meera Srivastava, Harvey B Pollard, Vijay K Singh

**Affiliations:** 1 Armed Forces Radiobiology Research Institute, Bethesda, MD, USA; 2 Department of Anatomy, Physiology and Genetics, and Center for Medical Proteomics, USA; 3 Department of Radiation Biology, F. Edward Hébert School of Medicine, Uniformed Services University of the Health Sciences, Bethesda, MD, USA

**Keywords:** Biomarkers, Gamma-radiation, Microarray, Proteomics, Stem cells, Tocopherol succinate

## Abstract

Tocopherol succinate (TS) has been shown to protect mice against acute radiation syndrome, however, its exact mechanism of action and its possible use in humans has not yet been evaluated. Our approach has been to test the radioprotectant properties of TS on CD34-positive stem cells from healthy volunteers. We hypothesize that a radioproteomics strategy can identify a drug-dependent, personalized proteomics signature for radioprotection. To directly test the radioproteomics hypothesis, we treated human CD34-positive stem cells with 20 μM TS for 24 h, and then exposed the cells to 2 Gy of cobalt-60 gamma-radiation. We isolated protein from all cultures and used a high throughput Antibody Microarray (AbMA) platform to measure concentrations of 725 low abundance proteins. As an in vivo control, we also tested mouse CD34-positive stem cells using the same preemptive TS paradigm on progenitor colony forming units. TS pretreatment of in vitro or in vivo CD34-positive stem cells rescued radiation-induced loss of colony-forming potential of progenitors. We identified 50 of 725 proteins that could be preemptively rescued from radiation-induced reduction by pretreatment with TS. Ingenuity Pathway Analysis (IPA) reveals that the modified proteins fall into categories dominated by epigenetic regulation, DNA repair, and inflammation. Our results suggest that radioproteomics can be used to develop personalized medicine for radioprotection using protein signatures from primary CD34-positive progenitors derived from the patient or victim prior to radiation exposure. The protective effect of TS may be due to its ability to preemptively activate epigenetic mechanisms relevant to radioprotection and to preemptively activate the programs for DNA repair and inflammation leading to cell survival.

## Introduction

The risk of exposure to ionizing radiation due to terrorist activities is widely thought to be increasing [1]. Although efforts to find suitable radiation countermeasures were initiated more than half a century ago, no safe and effective radiation countermeasure has been approved by the United States Food and Drug Administration (US FDA) for acute radiation syndrome (ARS). Thus, there is a pressing need to address this problem [2,3]. Major functional themes for countermeasure development have included free radical scavengers, hematopoietic progenitor stimulators, DNA repair enhancement, and blocking apoptotic pathways. Therapy with cytokine treatment and supportive care, which includes antibiotics and blood component transfusion, has shown limited success in animal models [3–5]. These results have prompted intensified research among government laboratories, academic institutions, and pharmaceutical companies to identify a new generation of countermeasures [2,3,6,7].

The biological effects of radiation are strongly dependent upon the dose of radiation received, and can result in hematopoietic, gastrointestinal (GI), and cerebrovascular syndromes of ARS [8,9]. There are a number of potential radiation countermeasures currently at different stages of development; these fall roughly into two categories depending upon their primary mechanism of action: immunomodulators/cytokines/growth factors [7] and antioxidants/free radical scavengers [3]. In large part, the focus on cytokines and growth factors has been based on their ability to enhance hematopoietic system recovery from radiation damage, demonstrated in multiple *in vitro* and *in vivo* systems [10]. Some cytokines have received FDA approval for treatment of neutropenia and thrombocytopenia caused by chemotherapy, and several others are under development [3]. The potential of antioxidants and free radical scavengers as radiation countermeasures is derived from their ability to reduce levels of radiation-induced reactive oxygen species, thus decreasing DNA damage, lipid peroxidation and other types of chemical damage [6].

There are eight distinct isomers of vitamin E, which are designated α, β, γ, and δ tocopherols and tocotrienols [11]. TS is the hemisuccinate ester of α-tocopherol. Earlier, we have demonstrated that TS protects mice against radiation-induced hematopoietic and GI syndromes, has a dose reduction factor of 1.28, and induces high levels of granulocyte-stimulating factor [12,13]. Blood response analysis has revealed that TS significantly reduces the severity of ionizing radiation-associated thrombocytopenia, neutropenia, and monocytopenia [14]. Furthermore, TS modulated antioxidant enzymes and oncogene expression leading to hematopoietic recovery [15]. Additionally, the protective effects of TS against radiation-induced hematopoietic and GI syndromes can be abrogated by the administration of a neutralizing granulocyte colony-stimulating factor antibody [12,16]. We have also demonstrated that TS mobilizes progenitors into the peripheral circulation, and that infusion of whole blood, or peripheral blood mononuclear cells, from TS-injected mice improves chances of extended survival of host mice exposed to radiation [12,17,18].

Recently, human CD34^+^ stem cells have been developed as a clinically relevant *in vitro* model for studying radioprotective efficacy of radiation countermeasures [17]. However, CD34^+^ cells come from healthy volunteers. We hypothesized that a radioproteomics strategy could identify a drug-dependent, *personalized* proteomics signature for radioprotection by preemptive administration of TS. To test this hypothesis, we exposed untreated and TS-treated CD34^+^ stem cells to ^60^Co- γ irradiation and determined that the radiation-dependent reduction of granulocyte/macrophage progenitor colony production could be rescued by pre-treatment of the cells with TS. Based on these positive results, we performed the following additional tests: (1) whether there were radiation-depressed proteins which could be rescued by TS pretreatment; and (2) whether the deduced radioproteomic signature had any relationship to the conventional molecular biology of radiation injury at the cellular level. We found positive results for both of these tests, and conclude that radioproteomics can be used to develop personalized medicine for radiation injury using protein signatures from primary CD34^+^ progenitors derived from the patient or victim prior to radiation exposure.

## Materials and Methods

### Mice

Male 6–8 week-old CD2F1 mice were purchased (Harlan Laboratories, Inc., Indianapolis, IN, USA) and housed (8 per cage) in a temperature and humidity controlled facility (21 ± 2°C, 50 ± 10% humidity) accredited by the Association for Assessment and Accreditation of Laboratory Animal Care-International. All mice were kept in rooms with a 12 h light/dark cycle and provided 10–15 hourly cycles of fresh air. Mice were provided certified rodent rations (Teklad Rodent Diet, Harlan Laboratories, Inc.) and acidified water (HCl, pH=2.5–2.8) *ad libitum*. Upon arrival, the mice were held in quarantine for one week. A microbiological examination of representative samples ensured the absence of *Pseudomonas aeruginosa*. All animal procedures were performed according to a protocol approved by the Armed Forces Radiobiology Research Institute’s (AFRRI) Institutional Animal Care and Use Committee. Research was conducted according to the Guide for the Care and Use of Laboratory Animals prepared by the Institute of Laboratory Animal Resources, National Research Council, US National Academy of Sciences [19].

### Cells

Human hematopoietic CD34^+^ cells from a 23 year old Caucasian male were procured from the National Hematopoietic Cell Processing Core, Fred Hutchinson Center Research Center (Seattle, WA, USA) [20]. Cells were cultured in serum-free medium consisting of Iscove’s Modified Dulbecco’s Medium (IMDM) supplemented with BIT 9500 (Stemcell Technologies Inc., Vancouver, BC, Canada), penicillin (100 U/ml; Invitrogen Corporation, Carlsbad, CA, USA), streptomycin (100 μg/ml; Invitrogen Corporation), recombinant human (rh) stem cell factor (SCF; 100 ng/ml; Stem cell Technologies Inc.), rh Fms like tyrosine kinase 3 (FLT3) ligand (100 ng/ml; Stemcell Technologies Inc.), and rh interleukin 3 (IL-3; 25 ng/ml; Stemcell Technologies Inc.). Culture flasks/dishes were incubated at 37°C with 5% CO_2_.

### Drug treatment

For *in vitro* experiments, TS (Sigma-Aldrich, St. Louis, MO, USA) stock solution was made by dissolving TS in dimethyl sulfoxide (DMSO; American Type Culture Collection, Manassas, VA) and stored at −20°C. TS stock solution was added to culture media to attain desired final concentration of TS. Final concentration of DMSO was 0.01% in media. Cells were treated with TS for 24 h and washed with media before irradiation. After irradiation, cells were again washed with fresh media. Cell survival and growth was tested over a period of time using MTS (tetrazolium compound [3-(4,5-dimethylthiazol-2-yl)-5-(3-carboxymethoxyphenyl)-2-(4-sulfophenyl)-2H-tetrazolium, Promega Corporation, Madison, WI, USA) assay. Mice were treated with TS as described earlier [12].

### Irradiation

Mice were placed in ventilated Plexiglas boxes compartmentalized to accommodate eight mice per box and exposed to midline dose of 11 Gy by bilateral irradiation in the AFRRI ^60^Co γ-radiation facility at a dose rate of 0.6 Gy/min. After irradiation, mice were returned to their cages and monitored. Radiation dosimetry was based primarily on the alanine/EPR (electron paramagnetic resonance) system [21,22], currently accepted as one of the most accurate methods and used for intercomparison between national metrology institutions. The details of dosimetry have been described earlier [23]. CD34^+^ cells were exposed to either 0 or 2 Gy (0.6 Gy/min) ^60^Co γ-radiation. Total surviving cells were counted using trypan blue (Sigma-Aldrich).

### Colony forming unit (CFU) assay with CD34^+^ cells

Colony forming unit-granulocyte/macrophage (CFU-GM) progenitors were assayed in semisolid cultures by culturing CD34^+^ cells as described earlier [24] with minor modifications [18]. The cells were resuspended and concentration was adjusted to 1×10^4^ cells/ml in MethoCult M3434 medium (Stemcell Technologies, Inc). A total of 300 μl of cell suspension was mixed gently with 3 ml of M3434 medium; and 1.1 ml of cell suspension was dispensed in 35 mm culture dishes in duplicate using a 16 gauge blunt-end needle. Three 35 mm dishes (two seeded with cells and one with sterile water) were placed into a 100 mm petri dish and incubated in 5% CO_2_ incubator at 37°C to score CFU-GM on day 14, along with a petri dish containing only sterile water.

### CFU assay with mouse bone marrow cells and CD34^+^ cells

Mice were humanely euthanized and the femur was collected at specified time after irradiation for bone marrow cells. CFU progenitors were assayed in semisolid media by culturing bone marrow cells as described earlier [18]. In brief, ends of the femur were trimmed to expose the interior marrow shaft. The marrow suspensions were treated with 2 ml of ammonium chloride at 4°C for 3 min to ensure the lysis of red blood cells and the suspension was enriched for bone marrow cells with the EasySep Mouse hematopoietic progenitor cell enrichment kit (Stemcell Technologies, Inc.) by following the protocol. Enriched bone marrow suspension was adjusted to a concentration of 1 × 10^8^ cells/ml in IMDM. Normal rat serum (50 μl/ml of cell suspension) was added to block the non-specific binding of lineage antibodies. EasySep Mouse hematopoietic progenitor cell enrichment cocktail, which contained biotinylated antibodies (CD5, CD11b, CD19, CD45R, 7–4, Ly-6G/C (Gr-1), TER119) (Stemcell Technologies, Inc.) directed against non-hematopoietic stem and progenitor cells was added (50 μl/ml of cell suspension) and the solution was incubated at 2 – 8°C for 15 min. EasySep Mouse progenitor magnetic microparticles (75 μl/ml of cells suspension) were added and allowed to incubate for 10 min. Then the cell suspension was exposed to a strong magnet for 3 min to segregate the unwanted cells. Enriched cell concentration was adjusted to 1 × 10^4^ cells/ml in MethoCult M3434 medium (Stemcell Technologies, Inc.). A total of 300 μl of cell suspension was mixed gently with 3 ml of M3434 medium; and 1.1 ml of cell suspension was dispensed in 35 mm culture dishes in duplicate using a 16 G blunt-end needle. Three 35 mm dishes (two seeded with cells and one with sterile water) were incubated at 37°C for 14 d. Burst forming unit erythroid (early erythroid precursors - BFU-E), CFU-GM, and multipotential progenitors (granulocyte, erythrocyte, monocyte, megakaryocyte, CFU-GEMM) were scored on 7 and 14 d [18,24].

## Protein Profiling using Antibody Microarrays

### Labeling of cell lysate

**Figure 1:**
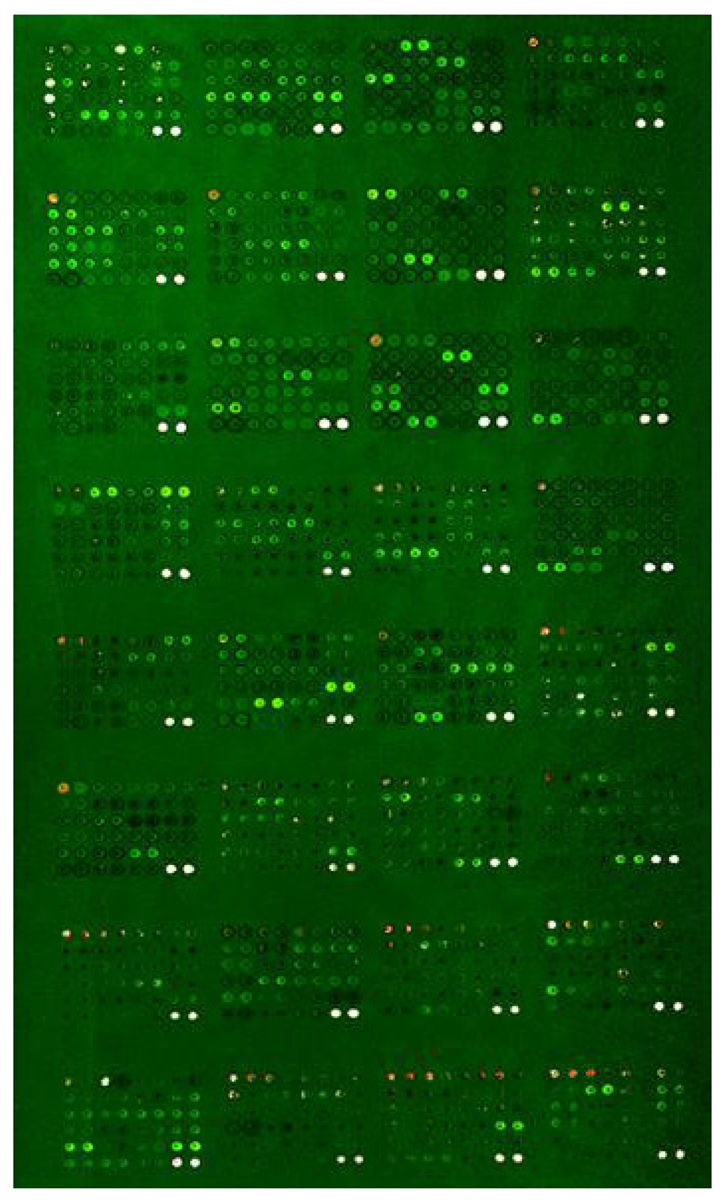
An example of an antibody array image of irradiated CD34^+^ cells vs reference standard. Proteins from irradiated or unirradiated CD34^+^ cells were labeled with Cy3 or Cy5 dyes and analyzed on an antibody microarray platform. Net green or red fluorescence indicate differences due to ^60^Co γ-irradiation. White spots indicate equivalent amounts of bound antigen.

Cell lysates were pooled on the basis of equal protein content and labeled with Cy3. Pooled cell lysate from 30 cell lines was labeled with Cy5 as an ‘internal standard’ and multiplexed with the Cy3-labeled experimental samples. Cy3-labeled pooled cell lysate was mixed with Cy5-labeled control on an equal volume basis. Each sample was incubated with a 725-feature antibody microarray (Panorama XPRESS Profiler 725, Sigma Aldrich) in a medium containing a detergent-based reagent to minimize protein–protein interactions as described previously [25]. Figure 1 shows an example of primary data in which irradiated and control CD34^+^ cells, labeled with either Cy3 or Cy5, were applied to the 725 feature antibody microarray platform.

### Fluorescence detection

The fluorescence at each spot on the antibody microarray was measured on a GenePix array reader (New Milton, New Hampshire, UK).

### Data quality control

Each cell lysate sample provided four replicate data points on the array. All spots with intensities below the local background or all spots with a signal-to-noise ratio <3 were rejected. We then calculated the average and standard deviation (SD) for each protein. Outliers were rejected if their deviations were larger than 2 SD’s from the average for each respective protein. The averages were then recalculated by omitting outliers. If the signal of a given protein was still too noisy, that specific protein was excluded from the analysis. We quantitated volume-normalized protein levels by ratioing Cy3-labeled proteins in cell lysate samples to the same protein, labeled with Cy5, in the normal control cell lysates. Normal cell lysates labeled with both Cy3 and Cy5 were ratioed to one another in order to calculate a labeling efficiency difference specific to each protein. Normalization according to the total protein was tested and excluded on the basis of profoundly noisy outcomes. Therefore, the protein levels defined by these assays are concentrations found in an untreated sample.

### Statistical analysis

The significant differences between treatment groups were determined by an analysis of variance (ANOVA) with a threshold *p* value of <0.05. A threshold *p* value of <0.01 was used for correlation analyses to indicate significant differences between treatment groups.

### Ingenuity Pathways Analysis

We used Ingenuity Pathway Analysis (IPA) software (Ingenuity Systems, Redwood City, CA, USA) to discriminate the molecular pathways responsible for irradiation effects versus TS protection. An average expression ratio R >2 in irradiated versus TS protection comparisons was used as the threshold. The reports with outlier proteins from the antibody microarray analysis were uploaded and mapped to corresponding objects (genes/proteins) in IPA’s database.

## Results

### Preemptive rescue of radiation-suppressed generation of immune and erythrocyte progenitor cells from CD34^+^ stem cells by TS

**Figure 2:**
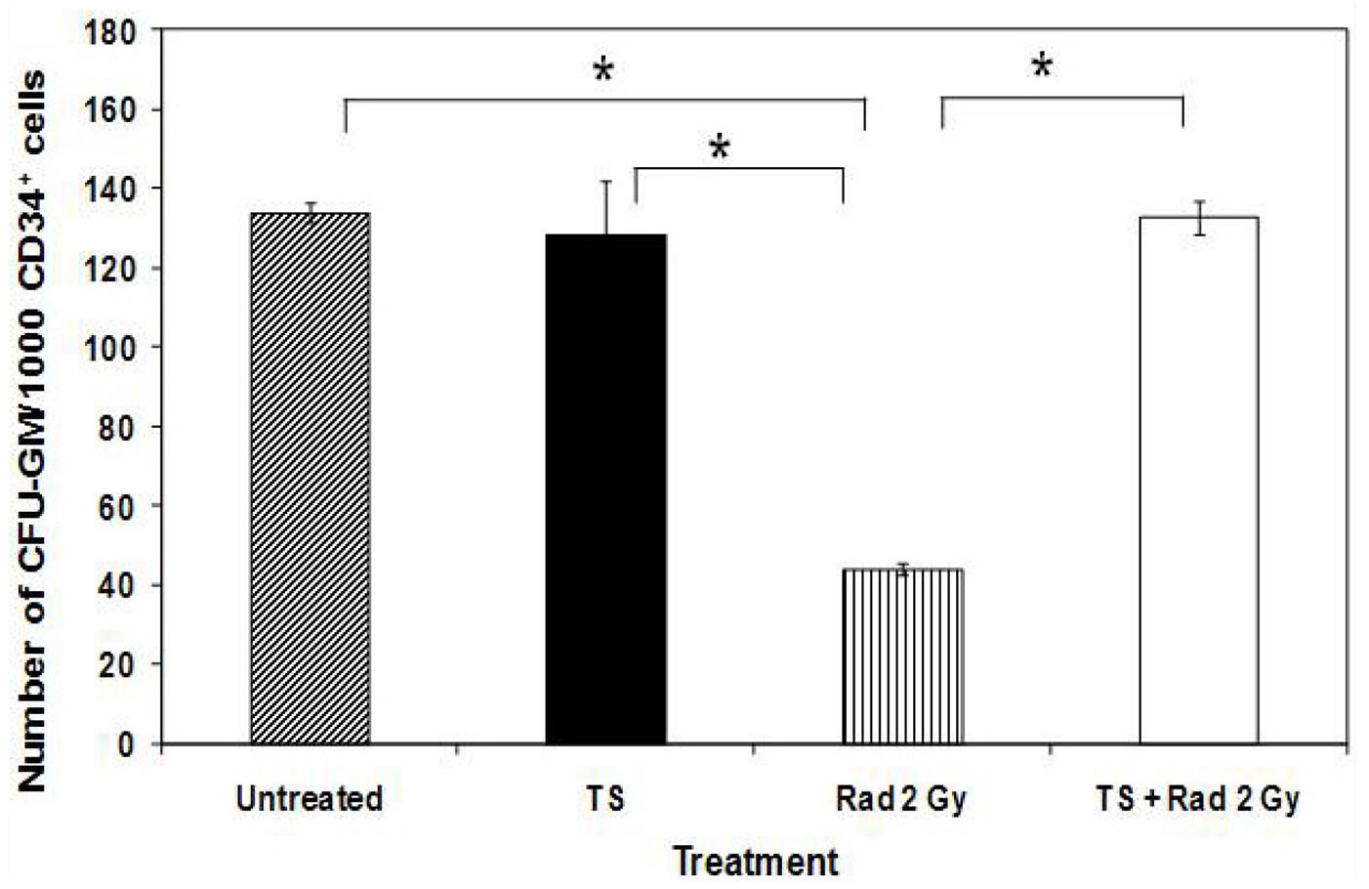
Effect of TS on CFU-GM colony count in irradiated CD34^+^ cells. CD34^+^ cells (5×10^5^ cells/ml) were treated with TS (20 μM) for 24 h, and irradiated (2 Gy, 0.6 Gy/min). Cells were diluted in Methocult H4535 medium (1,000 cells/ml) 24 h after irradiation and seeded in 33 mm petri dishes; CFU-GM colonies were scored on day 14 after irradiation.

Figure 2 shows that after 24 h of incubation, human CD34^+^ stem cells were able to generate *ca*. 130 CFU-GM/1000 cells (properties of stem cells). However, radiation treatment of the CD34^+^ stem cells significantly reduced GM progenitor colony forming activity by *ca*. 75%. Incubation with TS alone did not significantly modify the GM progenitor generation. However, treatment of the cells with TS for 24 h before irradiation rescued GM progenitor colony forming efficacy to levels statistically identical to unirradiated control. Thus TS prevented radiation suppression of CFU-GM in cultures of human CD34^+^ cells.

**Figure 3:**
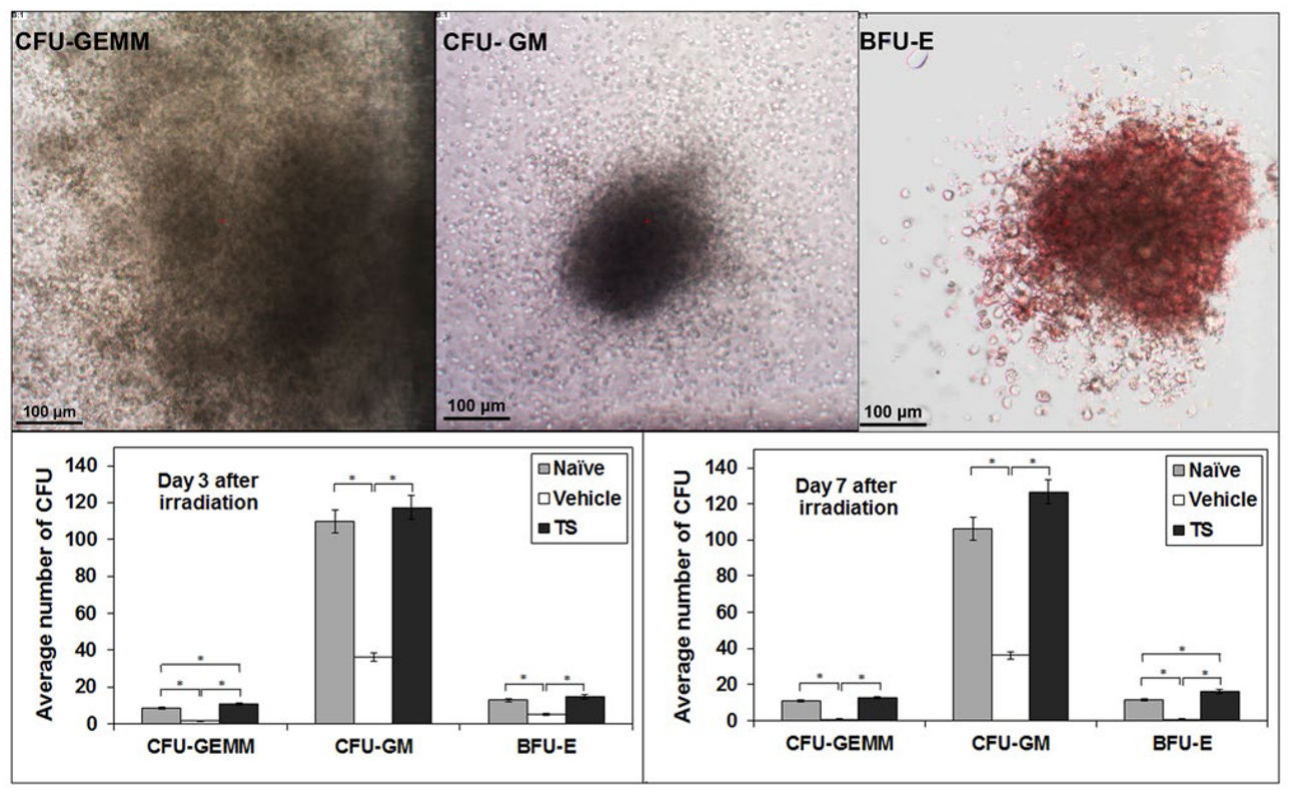
Effect of irradiation and TS treatment on bone marrow colony forming potential. Mice were treated with 400 mg/kg of TS or vehicle and irradiated 24 h after injection. Bone marrow cells were collected 3 and 7 d post-irradiation and the colonies of CFU-GEMM, CFU-GM, and BFU-E cells were counted. Representative photographs of colonies are shown (X400 magnification). *Denotes statistically significant difference between treatment groups *p*<0.05.

To test the *in vivo* effect, we treated mice with 400 mg/kg of TS, or vehicle, and irradiated the mice with 11 Gy of ^60^Co- γ irradiation. At 3 and 7 d after irradiation, we purified hematopoietic stem cells from bone marrow, and assayed for drug effects on radiation-induced loss of colony-forming potential for erythroid (E), GM, and GEMM progenitor cells. TS pretreatment resulted in increased levels of all three progenitor activities, with highest quantitative activity for CFU-GM progenitor cells (Figure 3). Thus, pretreatment of mouse with TS, *in vivo*, appears to support the *in vitro* radiation protection effect of pretreatment with TS of cultured human CD34^+^ cells.

### Identification of proteins that are altered by irradiation and for which change is prevented by TS pretreatment

**Figure 4:**
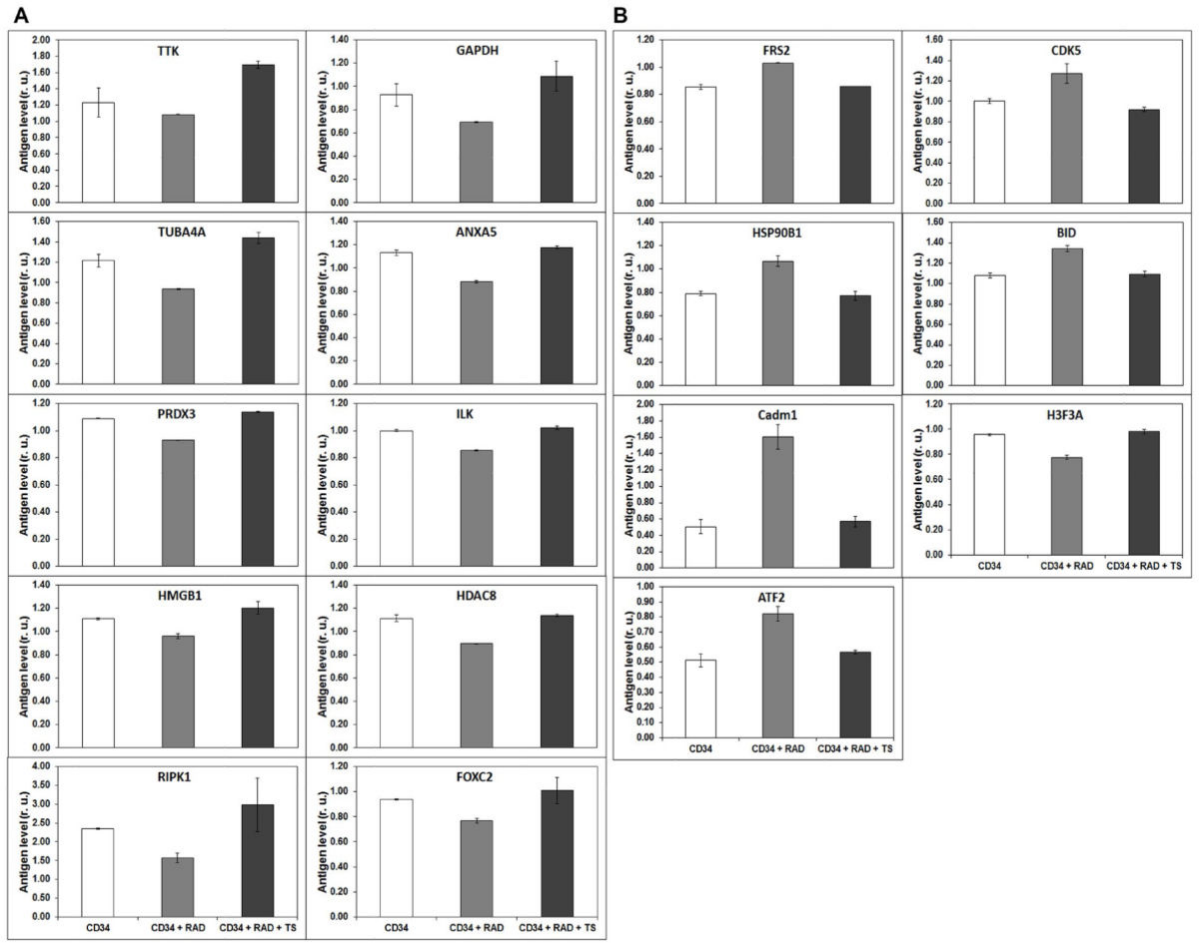
Effect of ^60^Co -irradiation and treatment of 20 μM TS on CD34^+^ cells. **A**. Proteins down-regulated by ^60^Co γ-radiation exposure and returned to control levels as a result of pretreatment with 20 μM TS. **B**. Proteins upregulated by ^60^Co γ-radiation exposure and returned to control levels as a result of pretreatment with 20 μM TS.

As shown in the Supplemental Table 1, irradiated CD34^+^ cells express a substantial number of proteins that are expressed quite differently from those in CD34^+^ cells treated before irradiation with TS. Here, we have specifically focused on a subset of top 30 proteins that are altered by radiation, and for which TS pretreatment returned them to normal levels. The rest of the proteins with less effect are also shown in the Supplemental Table 1. The top proteins down regulated by radiation are: TTK, TUBA4A, PRDX3, HMGB1, RIPK1, GAPDH, ANXA5, ILK, HDAC8, FOXC2. Pretreatment with TS prevented these proteins from down regulation when cells were irradiated (Figure 4A). By contrast, FRS2, CDK5, HSP90, BID, CADM1, H3F3A and ATF2 are up-regulated by γ-irradiation and TS pretreatment returned their expression to levels similar to those found in un-irradiated cells (Figure 4B). Protein expression changed as a result of irradiation and the TS treatment normalized the cells to the control state. Table 1 shows the percentage changes caused by irradiation and the percentage recovery when treated with TS.

Proteins involved in DNA repair and cell survival like **BP53, RIP1, PAD14 and DcR1** were down-regulated between 17% to 41% by irradiation, compared to the un-irradiated cells. Protein expression was recovered with TS pretreatment back to 87 to 128% of the values, from the un-irradiated cells. Proteins involved in transcriptional regulation like **HDAC8, HDAC2, and FOXC2** were down-regulated by 20% to 40% by irradiation and recovered with TS treatment to almost 78% to 107%. However, **ATF2** was up-regulated by 60.2% and TS treatment recovered its expression to 89.5% of the unirradiated value. Proteins involved in stress response, such as **hnRNAP1,** are down-regulated by 30% and recovered with TS treatment to 89.1%. Furthermore, PRDX3, involved in cell proliferation and differentiation, was down-regulated to 14.6% and recovered with TS treatment to 104.4%. **Cadm1** was upregulated 25.5% and recovered with TS treatment to 88.11%. These results suggest that the proteins involved in cell survival, DNA damage, DNA repair, and stress response, which are altered by irradiation, are brought back to normal levels with TS treatment.

### Ingenuity Pathway Analysis algorithm to analyze the TS-sensitive proteome in irradiated CD34^+^ stem cells

**Figure 5:**
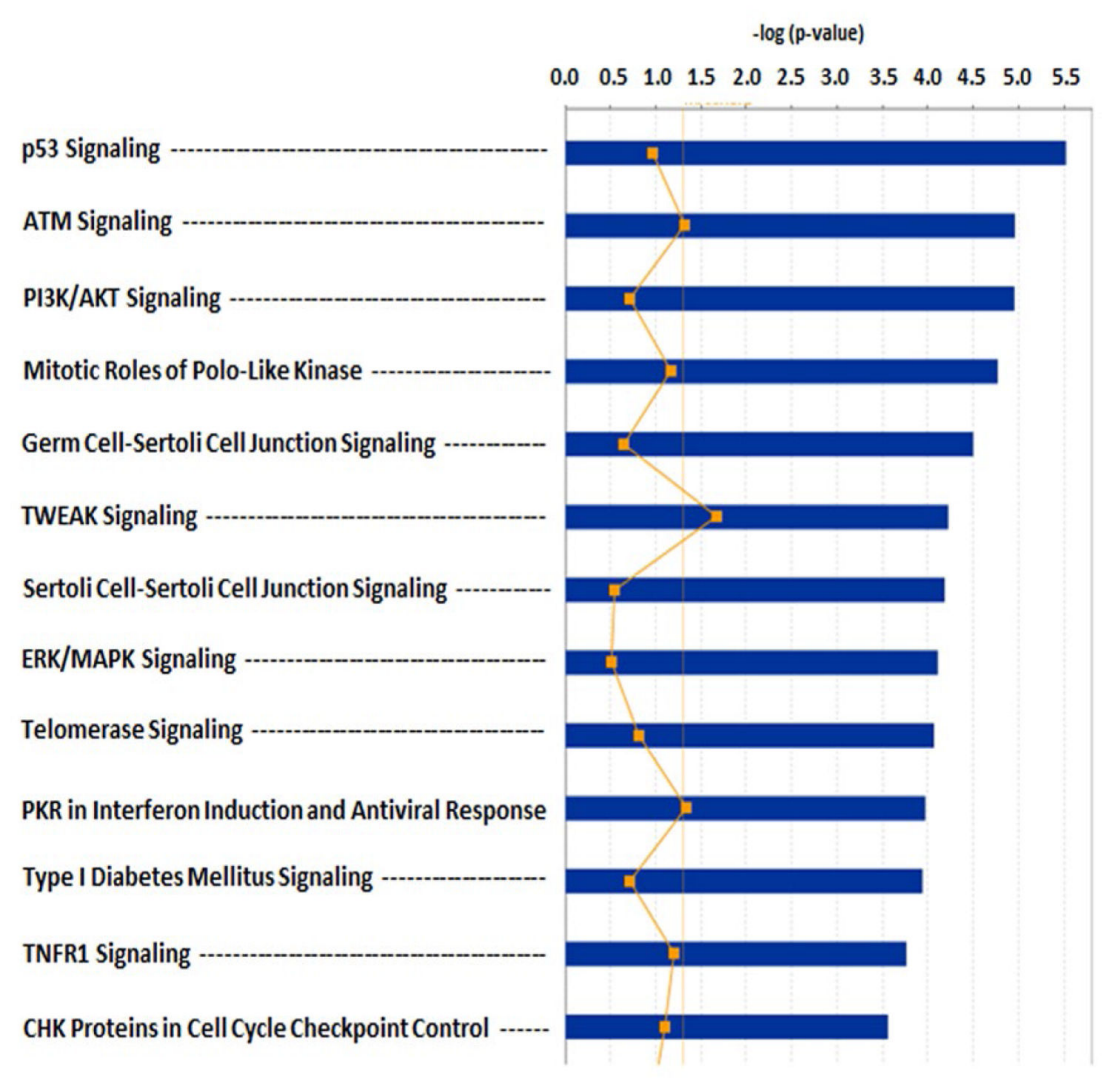
Gene ontology analysis of TS protection effects from irradiation on CD34^+^ progenitor cells. The principal TS effects are on *p53 signaling* (#1, p=*ca*. 10^−6^), *ATM signaling* (#2, p=*ca*. 10^−5^) and PI3K/AKT (#3, p=*ca*. 10^−5^).

The proteomic profile obtained was analyzed to identify pathways involved in the protective mechanism activated by TS. To elucidate their biological functions and to help uncover the mechanism of TS radioprotection, functional enrichment analysis was also carried out for the differentially expressed proteins. The initial IPA analysis in Figure 5 indicates that the TS affects *p53 signaling* (p=*ca*. 10^−8^), *ATM signaling* (p=*ca*. 10^−7^), and PI3K/AKT (p=*ca*. 10^−6^) signaling. IPA mapped all the differentially expressed proteins by specific molecular functions, biological processes and cellular components. Significant enrichment clusters of diseases such as cancer (p=*ca*. 10^−8^), and inflammation (p=*ca*. 10^−6^), functions such as cell death and survival (p=*ca*. 10^−16^), and physiological processes such as embryonic development (p=*ca*. 10^−9^) emerged from this analysis (Table 2).

**Figure 6:**
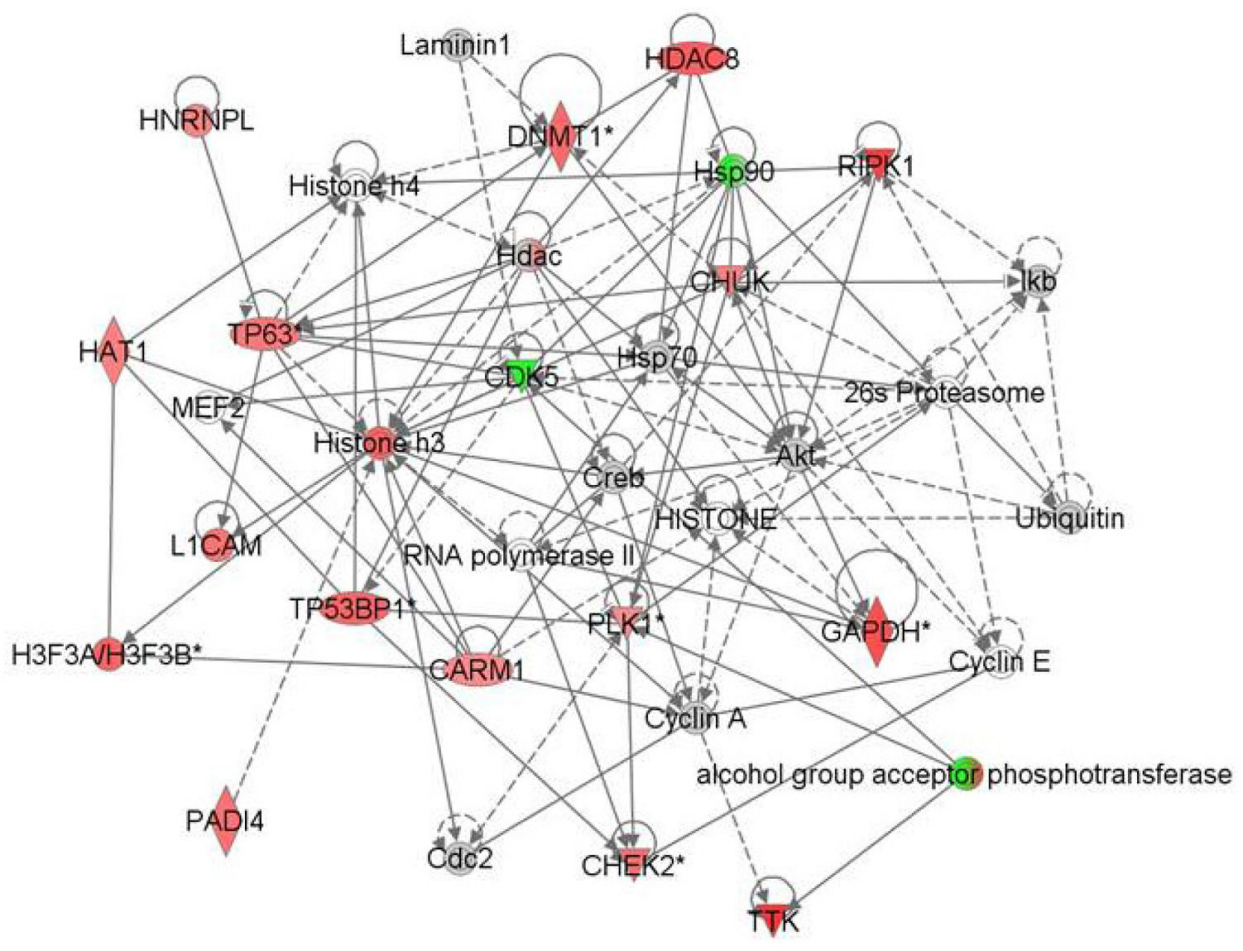
Ingenuity Pathway Analysis of TS protective effects from irradiation on CD34^+^ progenitor cells. The network was created using Path Designer (Ingenuity Pathways Analysis). Of the 50 top differentially expressed proteins that are protected by TS, 41 proteins are up-regulated (red color) and 9 were down-regulated (no color) by TS and are found in the set of proteins significantly (*p*<0.05) affected by TS.

To further understand the radioprotective effects of TS on CD34^+^ cells, we used the IPA software to identify the top network of the most affected protein biomarkers. Figure 6 shows the predicted relationships between the most affected proteins in color and the molecules that have been shown to be functionally connected. The pathways that are potentially controlled by the TS regulated biomarkers; many are associated with cancer, DNA repair and cell survival.

## Discussion

Radioproteomics provides a “big-data”-centric solution to the problem of identifying inherited variation in the mechanisms of response to radiation, susceptibility to radiation injury, and efficacy of radioprotective agents. The field of radiogenomics has recently been proposed for the purpose of developing personalized medicine for humans exposed to radiation [26]. However, candidate gene studies based on single nucleotide polymorphisms have not yet been able to explain a significant fraction of patient-to-patient variability in radiation response [27,28]. The problem with this nucleic acid-centric approach is that gene expression is sensitive to epigenetic regulation, as further emphasized in the present proteomic analysis. Another problem is that mRNA translation into protein is regulated by cytosolic mechanisms, including epigenetic microRNA mechanisms. Thus, while the nucleus “proposes” possible protein expression via mRNA and microRNA expression, the cytosol “approves” which proteins are to be expressed, and to what extent. The work described here shows that radioproteomics can bypass these potential radiogenomic problems by moving directly to protein control. Furthermore, using primary CD34^+^ stem cells from a healthy volunteer as a target for radioproteomic analysis, we are able to unambiguously identify a personalized radioproteomics mechanism for radioprotection by TS. Other candidate agents can be studied to determine their unique radioproteomics signature. Inasmuch as CD34^+^ stem cells can be routinely isolated as primary “explants” from patients, this radioproteomics strategy can be widely deployed in local clinical settings for personalized radiation medicine.

### Radioproteomics and its application to the mechanism of TS

We hypothesized that the cellular environment would change after irradiation and treatment with TS, thereby altering protein expression of CD34^+^ progenitor cells. Furthermore, we hypothesized that these expression changes could be detected and used to create a set of biomarkers associated with radioprotection.

**Figure 7:**
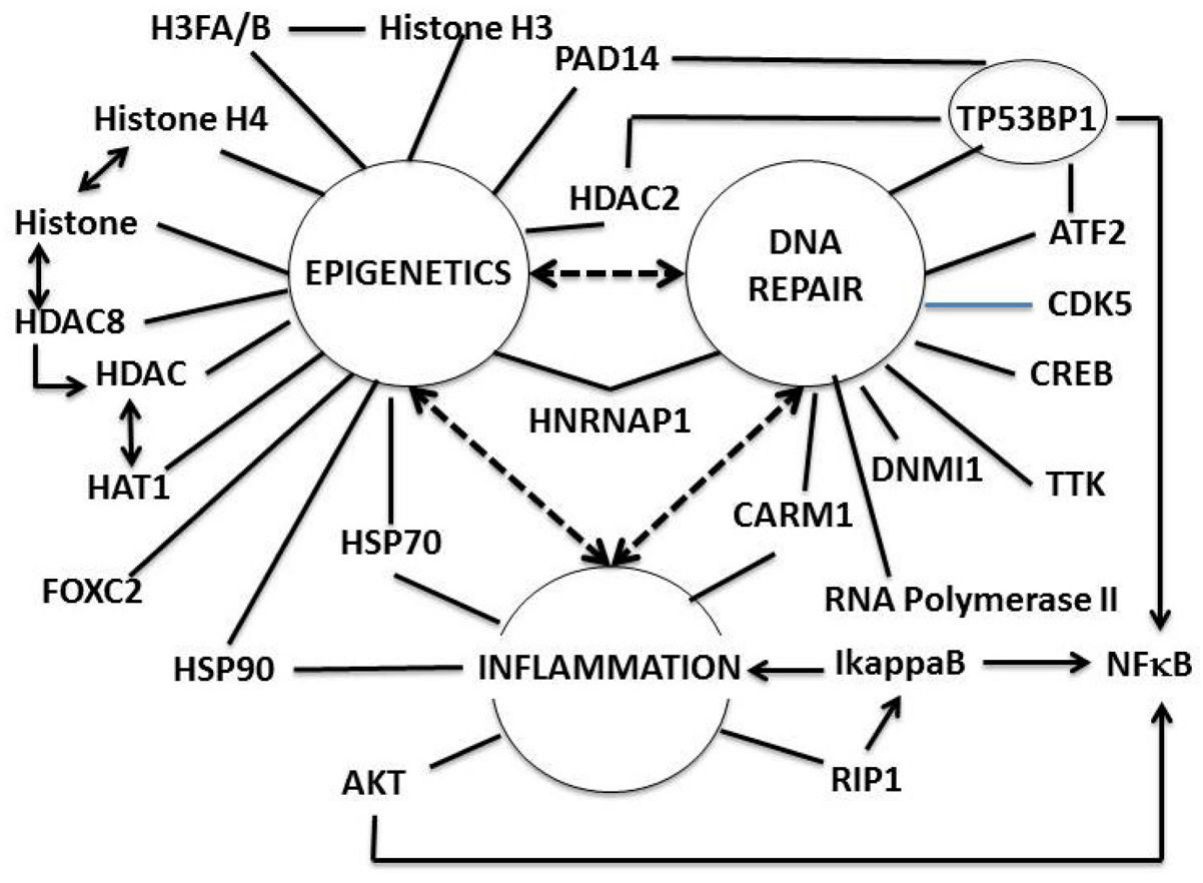
Radioproteomics of TS. Bioinformatics analysis shows that at least three primary hubs are able to provide an armature for the principal proteins associated with the radioproteomic signature of TS. These are epigenetics (13 spokes), inflammation (six spokes), and DNA repair (9 spokes). Furthermore, according to literature referred to in the text, all three hubs are connected functionally (heavy bi-tipped arrows), at least 5 proteins are shared by at least two hubs.

The hub-and-spoke analysis shows that epigenetic regulation is a highly populated hub for proteins associated radiation injury, which can be prevented by TS pretreatment, before irradiation in CD34^+^ stem cells (Table 3, Figure 7). The emphasis on epigenetic mechanisms suggests that TS is able to preemptively modify gene expression or putatively wildtype/“normal” genes that would otherwise be suppressed by radiation exposure. These proteins are involved in DNA repair and inflammation. The changes in epigenetic regulation induced by radiation and preempted by TS may provide some general insight into the singular lack of success for the radiogenomics search for alleles with altered sequences that might predispose individuals to mild or severe radiation injury. Epigenetic regulation can alter levels of mRNAs and microRNAs, and therefore cognate proteins, without an actual change in gene sequence. More recently chromatin regulation has been shown to play a critical role in hematopoietic stem cells, and genome-wide chromatin reorganization has been documented in the process of maturation [29]. Secondly, the expression levels of many mRNAs, and therefore their cognate proteins, are inherited [30]. However, the relationship between mRNA and protein levels is not perfect, due to the many post-transcriptional and post-translational regulatory mechanisms. Thus, a radioproteomics strategy might be expected to yield the most actionable information in terms of specific proteins, and even provide suggestions to where to look in whole genome sequences for radiogenomics information. Importantly, the present study is not without limitations. Given the focus on a single CD34^+^ stem cell biopsy, the study only provides proof-of-principal for the radioproteomics concept; many more studies will be needed, not only with additional “healthy controls”, but also on CD34^+^ stem cells from donors who are on a therapeutic trajectory. Studies will also be needed to determine the influence of age, gender, ethnicity, family health history and other demographic elements.

### Suitability of CD34^+^ stem cells as subjects of personalized radio-proteomic analysis

CD34^+^ progenitor cells are multipotential hematopoetic stem cells which can differentiate into the entire hematopoetic lineage [31–37]. Consequently, autologous CD34^+^ stem cells can be administered therapeutically to cancer patients following radiation-dependent bone marrow ablation, thereby successfully transplanting an entire cancer-free hematopoetic lineage [38]. In addition, the CD34^+^ cells may include angiogenic progenitors. These are being developed to treat peripheral, myocardial and cerebral ischemia [39]. A culture of CD34^+^ stem cell has been included in the ENCODE database, along with many other traditional cultured cells [40]. However, a CD34^+^ cell culture is actually a primary “biopsy” that directly represents the biology of the donor immune system on or about the day of collection. Therefore, by using a specific primary culture of CD34^+^ cells to evaluate a potential radioprotective agent [41,42], the experiment is actually identifying a personalized radioproteomic signature that is specific to the individual who donated the CD34^+^ cells. Cultures of CD34^+^ stem cells can be isolated non-invasively from anyone at any time. We suggest that CD34^+^ cells may be viewed as “low hanging fruit” for development of personalized radiation medicine, or other medical indications affected by mutation or environmental hazard.

## Supplementary Material

Supplementary table

## Figures and Tables

**Table 1:** Proteins with significantly different expression between irradiation and TS treatment in CD34^+^ progenitor cells.

Down Regulated Proteins in IR			Up-Regulated Proteins in irradiated cells		
Protein	% Change	% of recovery with TS Treatment	Protein	% Change	% of recovery with TS Treatment
DcR1	40.40	87.80	ATF2	60.19	89.51
HDAC2	39.85	78.26	HSP90B1	35.00	103.58
RIP1	33.09	127.00	H3F3A	29.58	99.06
hnRNAP1	30.00	89.09	Cadm1	2 15.88	88.11
PAD14	23.07	98.88	BID	24.34	98.62
HDAC8	19.52	102.11			
FOXC2	18.08	107.00			
BP53	17.49	128. 60			
PRDX3	14.57	104.40			

Data is presented as the percent change in protein expression, after exposure to 2 Gy γ-radiation and TS treatment, in relation to the values of non-irradiated cells.

**Table 2:** The top three biofunction categories by GO analysis.

Diseases and Disorders	*p*-value
Cancer	7.25E -08–2.11E-03
Inflammatory Response	1.24E -06–1.91E-03
Skeletal and Muscular Disorders	1.39E -06–1.59E-03
Connective Tissue Disorders	2.25E -06–9.72E-04
Inflammatory Disease	2.25E -06–9.72E-04
Molecular and Cellular Functions	*p*-value
Cell Death and Survival	6.34E-16–2.06E-03
Cell Cycle	1.37E-15–2.07E-03
Cellular Assembly and Organization	3.34E-10–1.75E-03
Cellular Function and Maintenance	3.34E-10–2.01E-03
Cellular Growth and Proliferation	2.12E-09–2.15E-03
Physiological System Development and Functions	*p*-value
Organism Survival	5.93E-10–1.56E-03
Embryonic Development	5.86E-09–1.88E-03
Organ Development	5.86E-09–1.88E-03
Organismal Development	5.86E-09–1.96E-03

Shown are enrichment clusters of diseases such as cancer (p=*ca*. 10^−8^), and inflammation (p=*ca*. 10^−6^), molecular functions such as cell death and survival (p=*ca*. 10^−16^), and physiological processes such as organismal survival (p=*ca*. 10^−10^) and embryonic development (p=*ca*. 10^−9^).

**Table 3:** Hub and spoke analysis for radioproteomics of TS effects on CD34^+^ stem cells.

HUBS	SPOKES
HISTONE H3	16
AKT	15
HSP90	12
CDK5	12
CREB	10
HSP70	10
HDAC	10
26S proteosome	10
CHUK	10
RNA Polymerase II	9
CYCLIN A	9
DNMI1	8
HISTONE H4	8
CARM1	7
TP53BP1	6
Histone	6
IkappaB	6

## Abbreviations

AbMA: Antibody Microarray
AFRRI: Armed Forces Radiobiology Research Institute
APP: Amyloid Precursor Protein
ARS: Acute Radiation Syndrome
ATF2: Activating Transcription Factor-2
ATM: Ataxia Telangiectasia Mutated
BFU-E: Burst Forming Unit Erythroid
Cadm1: Cell Adhesion Molecule-1
CD5: Cluster Of Differentiation-5
CD11b: Cluster Of Differentiation-11b
CD19: Cluster Of Differentiation-19
CD45R: Cluster Of Differentiation-45R
CFU-GM: Colony Forming Unit-Granulocyte/Macrophage
CFU-GEMM: Colony Forming Unit-Granulocyte Erythrocyte/Monocyte Megakaryocyte
Cy3: Cyanine-3
Cy5: Cyanine-5
DMSO: Dimethyl Sulfoxide
DNMT1: DNA (Cytosine-5)-Methyltransferase 1
E: Erythroid
ENCODE: Encyclopedia of DNA Elements
EPR: Electron Paramagnetic Resonance
FLT3: Fms like Tyrosine Kinase 3
FOXC2: Forkhead Box Protein C2
GEMM: Granulocyte Erythrocyte Monocyte Megakaryocyte
GI: Gastrointestinal
GM: Granulocyte Macrophage
GO: Gene Ontology
HDAC2: Histone Deacetylase 2
HDAC8: Histone Deacetylase 8
hnRNPA1: Heterogeneous Nuclear Ribonucleoprotein A1
hnRNPU: Heterogeneous Nuclear Ribonucleoprotein U
IL-3: Interleukin 3
IMDM: Iscove’s Modified Dulbecco’s Medium
IPA: Ingenuity Pathway Analysis
MTS: Tetrazolium Compound [3-(4,5-dimethylthiazol-2-yl)-5-(3-carboxymethoxyphenyl)-2-(4-sulfophenyl)-2H-tetrazolium
OP18: Oncoprotein18
p53: Protein 53
PADI4: Peptidyl Arginine Deiminase, type IV
PI3K/AKT: Phosphoinositide 3-Kinase/Protein Kinase B
PRDX3: Peroxiredoxin 3
qPTL: Protein Quantative Train Loci
rh: Recombinant Human
RIP1: Receptor-Interacting Protein 1
SD: Standard Deviation
TBP: TATA-Binding Protein
