# Supplementary material for: Personalized Radioproteomics: Identification of a Protein Biomarker Signature for Preemptive Rescue by Tocopherol Succinate in CD34+ Irradiated Progenitor Cells Isolated from a Healthy Control Donor
Source: J Proteomics Bioinform. Author manuscript; Available in PMC 2016 Apr 15. (PMC4833407; doi:10.4172/jpb.1000349)
Supplement: Supplementary table [file NIHMS765570-supplement-Supplementary_table.docx]

Supplemental Table 1. Proteins with significantly different expression between irradiation and TS treatment in CD34^+^ progenitor cells.

|  | | **Log (normalized intensity)** | | |
| --- | --- | --- | --- | --- |
| **Symbol** | **Antigen** | **CD34 cells** | **CD34 + Rad** | **CD34 + TS + Rad** |
| TTK | hMps1 | 1.23 | 1.08 | 1.69 |
| TUBA4A | Tubulin Polyglutamylated | 1.22 | 0.94 | 1.44 |
| PRDX3 | Peroxiredoxin 3 | 1.09 | 0.93 | 1.14 |
| HMGB1 | HMG1 | 1.11 | 0.96 | 1.20 |
| RIPK1 | RIP Receptor Interacting Protein | 2.35 | 1.57 | 2.98 |
| GAPDH | GAPDH | 0.93 | 0.69 | 1.09 |
| ANXA5 | Annexin V | 1.13 | 0.88 | 1.18 |
| ILK | ILK | 1.00 | 0.86 | 1.02 |
| HDAC8 | HDAC8 | 1.11 | 0.90 | 1.14 |
| FOXC2 | FOXC2 | 0.94 | 0.77 | 1.01 |
| H3F3A | Histone H3 pSer10 | 0.96 | 0.77 | 0.98 |
| KRT18 | Cytokeratin peptide 18 | 0.85 | 0.59 | 0.87 |
| TP53BP1 | P53 BP1 | 1.21 | 1.00 | 1.56 |
| DDX20 | Gemin3 | 0.94 | 0.80 | 0.96 |
| DNMT1 | DNMT1 | 1.31 | 1.14 | 1.30 |
| FRS2 | FRS2 | 0.85 | 1.03 | 0.86 |
| MAPK14 | p38 MAP Kinase NonActivated | 1.19 | 1.05 | 1.28 |
| CETN1 | Centrin | 1.58 | 1.08 | 1.64 |
| INA | aInternexin | 1.05 | 0.86 | 1.17 |
| CHEK2 | Chk2 | 1.07 | 0.84 | 1.07 |
| L1CAM | L1CAM | 0.99 | 0.83 | 1.07 |
| PADI4 | PAD14 | 1.10 | 0.85 | 1.09 |
| DMD | Dystrophin | 1.03 | 0.89 | 1.03 |
| BCAR1 | p130CAS | 0.93 | 0.76 | 0.92 |
| Ksr1 | KSR | 1.05 | 0.90 | 1.09 |
| TP63 | p63 | 1.13 | 0.82 | 1.12 |
| CDK5 | CDK5 | 1.00 | 1.27 | 0.92 |
| SYNPO | Synaptopodin | 1.04 | 0.88 | 1.06 |
| CASP4 | Caspase 4 | 1.15 | 0.91 | 1.12 |
| FOXP2 | FOXP2 | 1.06 | 0.89 | 1.03 |

The table is sorted by the most affected proteins by radiation and recovery by TS.
